# MolluscDB: a genome and transcriptome database for molluscs

**DOI:** 10.1098/rstb.2020.0157

**Published:** 2021-05-24

**Authors:** Carlos Caurcel, Dominik R. Laetsch, Richard Challis, Sujai Kumar, Karim Gharbi, Mark Blaxter

**Affiliations:** ^1^ Institute of Evolutionary Biology, School of Biological Sciences, University of Edinburgh, Edinburgh EH9 3JT, UK; ^2^ Edinburgh Genomics, School of Biological Sciences, University of Edinburgh, Edinburgh EH9 3JT, UK; ^3^ Tree of Life Programme, Wellcome Sanger Institute, Wellcome Genome Campus, Hinxton, Cambridge CB10 1SA, UK

**Keywords:** molluscs, database, genome, transcriptome, protein families, shell matrix proteins

## Abstract

As sequencing becomes more accessible and affordable, the analysis of genomic and transcriptomic data has become a cornerstone of many research initiatives. Communities with a focus on particular taxa or ecosystems need solutions capable of aggregating genomic resources and serving them in a standardized and analysis-friendly manner. Taxon-focussed resources can be more flexible in addressing the needs of a research community than can universal or general databases. Here, we present MolluscDB, a genome and transcriptome database for molluscs. MolluscDB offers a rich ecosystem of tools, including an Ensembl browser, a BLAST server for homology searches and an HTTP server from which any dataset present in the database can be downloaded. To demonstrate the utility of the database and verify the quality of its data, we imported data from assembled genomes and transcriptomes of 22 species, estimated the phylogeny of Mollusca using single-copy orthologues, explored patterns of gene family size change and interrogated the data for biomineralization-associated enzymes and shell matrix proteins. MolluscDB provides an easy-to-use and openly accessible data resource for the research community.

This article is part of the Theo Murphy meeting issue ‘Molluscan genomics: broad insights and future directions for a neglected phylum’.

## 1. Introduction

Molluscs are important members of many terrestrial and aquatic ecosystems, where they can be dominant herbivores or important predators. They are important indicators of ecosystem health and are highly responsive to environmental change. Molluscs are also major food sources for humans, and understanding their biology is important for improving shellfish productivity. The mollusc shell is a product of active biomineralization, the enzymology of which is of interest to synthetic biology, aquaculture and climate science. Improved understanding of all of these issues and questions can be gained through the application of genomic and post-genomic methodologies to target species of molluscs, and through comparison of mollusc genes and genomes.

Over the past years, sequencing costs have decreased significantly. As a result, sequencing projects are no longer limited to specialized centres or large consortia [1]. This has favoured the study of non-model organisms such as molluscs, for which the number of sequencing-related publications is rapidly growing [2]. During the publication process, raw data and draft genomes are submitted to public databases. However, other analysis outputs such as gene predictions or functional annotations are often not formally submitted, which hinders further analyses by other researchers. Exceptionally, research groups make their results publicly available through their own websites, but such resources can be hard to maintain, tend to increase format inconsistency between projects and do not offer a stable environment for the exploitation of the data. The situation is worse for species for which only transcriptome data are available, as there is no widely accepted route for submission of transcriptome assembly and annotation data for reuse. Usually, transcriptome data are only available in the form of raw reads, and reuse of these data often requires reassembly and reanalysis, divorcing secondary use from the primary publication [3,4].

While pan-taxonomic databases such as Ensembl [5] host and maintain data in a reliable way and provide a diverse set of tools for data exploration, they cannot accommodate most datasets generated in genomic research owing to strict quality inclusion criteria. For model organisms, data are hosted in taxon-specific databases, such as FlyBase [6] or WormBase [7]. These offer a feature-rich presentation and analysis ecosystem that can focus on meeting the specific needs of their research communities. Owing to their multiple advantages, taxon-specific databases have also been developed for non-model organisms, such as VectorBase [8], Avianbase [9] and Lepbase [10]. This tiered system of databases, from Tier 1 (local, often private, single-species or single-analysis resources) through Tier 2 (multi-species databases) and Tier 3 (pan-species databases such as Ensembl) [11] delivers to the needs of the science community. However, tools for Tier 2 databases that integrate genome and transcriptome data are lacking.

MolluscDB is a Tier 2 genome and transcriptome database for the phylum Mollusca built with GenomeHubs [12]. MolluscDB delivers the core elements of Ensembl, plus additional resources conferred by GenomeHubs. Here, we discuss the features of MolluscDB and highlight its utility by using it to support analyses of mollusc phylogeny, of gene family evolution and of genes associated with biomineralization.

## 2. Methods

### (a) Software tools used

Relevant parameters and version numbers of software tools are given in electronic supplementary material, table S5.

### (b) Infrastructure

MolluscDB consists of a set of Docker containers and scripts provided by GenomeHubs. Infrastructural containers (EasyMirror [12], SequenceServer [13], h5ai (https://larsjung.de/h5ai/) and MySQL) were set up on an LXC container running Ubuntu 18.04 on a dedicated server (with access to 4 cores and 16 GB of RAM). Analytical and import-related containers (BLAST [14], BUSCO [15], CEGMA [16,17], InterProScan [18], RepeatMasker [19] and EasyImport [12]) were run when needed in a local compute cluster at the Institute of Evolutionary Biology, Edinburgh (768 cores, 384 GB to 1.5 TB RAM per node). Certain features of the Ensembl instance were modified using a custom plugin (https://github.com/genomehubs/molluscdb-plugin/).

### (c) *De novo* transcriptome assembly

Raw reads from different studies were downloaded from the Sequence Read Archive (SRA) [20]. The quality of each dataset was first assessed with FastQC [21]. Raw reads were trimmed to remove adapters and low quality or very short sequences with BBDuk [22]. Trimmed reads were then *de novo* assembled with Trinity [23]. Finally, candidate coding regions within the transcripts were predicted with TransDecoder [24]. Flags to account for strand specificity of the datasets were used for both Trinity and TransDecoder when appropriate. BLAST searches against UniRef90 [25] and hmmscan [26] searches against Pfam [27] were included in the TransDecoder pipeline to maximize sensitivity for capturing open reading frames.

### (d) Data import

Transcriptome and genome assemblies from different sources were incorporated into the database (table 1 and electronic supplementary material, table S1). Three species were directly mirrored from Ensembl Genomes [28–31]. Four genome assemblies were imported together with their proteins and gene models from ngenomes.org [32], VectorBase [8,33] and the NCBI Assembly resource [34,35]. Fifteen transcriptome assemblies were added to the database: 4 published assemblies [36–38] and 11 transcriptomes that were *de novo* assembled from publicly available raw read data [3,39–44]. For all the transcriptomes, proteins were predicted and imported together with their assemblies.

**Table 1.** Genome and transcriptome assemblies available in MolluscDB^a^. An extended version of this table is available in electronic supplementary material, table S1.

species	class	span (Mb)	scaffold count	scaffold N50 (kb)	contig count	contig N50 (kb)	BUSCO complete (%)^b^
genome data							
*Bathymodiolus platifrons*	Bivalvia	1658	65 662	343	272 497	13	85.80
*Crassostrea gigas*	Bivalvia	558	7658	402	30 459	31	94.10
*Modiolus philippinarum*	Bivalvia	2630	74 573	100	301 873	39	82.60
*Octopus bimaculoides*	Cephalopoda	2338	151 674	475	700 124	6	86.80
*Biomphalaria glabrata*	Gastropoda	916	331 400	48	369 696	13	84.50
*Lottia gigantea*	Gastropoda	360	4469	1870	18 335	96	94.40
*Lymnaea stagnalis*	Gastropoda	997	148 229	5	328 378	6	88.10
*Capitella teleta (outgroup)*^a^	Polychaeta	277	20 803	188	49 393	22	98.40
transcriptome data							
*Cristaria plicata*	Bivalvia	418			523 239	2	100.00
*Laternula elliptica*	Bivalvia	297			324 119	1	98.60
*Mya arenaria*	Bivalvia	76			118 239	1	70.30
*Mya truncata*	Bivalvia	361			684 686	1	96.70
*Mytilus edulis*	Bivalvia	336			592 134	1	84.80
*Mytilus galloprovincialis*	Bivalvia	181			227 675	1	92.10
*Pecten maximus*	Bivalvia	195			298 288	1	85.80
*Scutopus ventrolineatus*	Caudofoveata	116			246 430	1	72.90
*Octopoteuthis deletron*	Cephalopoda	114			122 672	2	95.70
*Vampyroteuthis infernalis*	Cephalopoda	105			149 961	1	88.80
*Laevipilina hyalina*	Monoplacophora	135			287 179	1	52.50
*Acanthochitona crinita*	Polyplacophora	208			266 385	1	96.70
*Gadila tolmiei*	Scaphopoda	181			345 172	1	93.70
*Gymnomenia pellucida*	Solenogastres	194			266 289	1	86.10
*Wirenia argentea*	Solenogastres	327			563 852	1	73.00

^a^*Capitella teleta* is not a mollusc so is not present in the database, which is why it is marked as ‘outgroup’.

^b^BUSCO loci from the eukaryota_odb9 set identified in the assembly.

### (e) GenomeHubs analyses

During importation of genome or transcriptome data, GenomeHubs performs a number of analyses. Genome assemblies were masked with RepeatMasker [19] using the built-in Metazoa repeat database. Sequence similarity of the proteins to Swiss-Prot [25] was determined with BLAST [14]. Domain annotation and GO terms were obtained for each protein via InterProScan [18]. Genome completeness was evaluated with CEGMA [16,17] and BUSCO [15]. The same analyses were performed on transcriptome assemblies except for CEGMA and RepeatMasker.

A full set of orthology predictions and gene tree reconstructions was performed to enable Ensembl Compara [45] data displays within MolluscDB. The full orthology pipeline used Orthofinder 2 [46] for the majority of analysis steps and was implemented in a GenomeHubs Compara container [12]. Protein sequences in MolluscDB were clustered based on pairwise DIAMOND [47] blastp searches using the default inflation parameter of 1.5. For each orthogroup, protein sequences were aligned using MAFFT [48] and the resulting alignments were trimmed to remove poorly aligned regions using trimAl [49]. Approximate maximum-likelihood gene trees were reconstructed using FastTree [50] and reconciled against a species tree generated during the same Orthofinder run from a concatenated alignment of 1163 single-copy or mostly single-copy genes to identify gene duplication events, orthologs and paralogs. Orthofinder results were processed and imported into a Compara database as part of the GenomeHubs import.

### (f) Analysis of protein families

Protein, GFF3 and InterProScan files for the 22 species of molluscs detailed in table 1 were downloaded from MolluscDB via the download section. As two gene sets were available for *Lymnaea stagnalis*, only the files corresponding to the AUGUSTUS [51] annotation were used. Additionally, protein and GFF3 files of the annelid *Capitella teleta* were downloaded from Ensembl Genomes [28,31], and InterProScan [18] annotation was generated using the same version and parameters used for the MolluscDB species. Protein files were filtered to remove sequences shorter than 30 residues and predicted peptides with internal stop codons. For transcriptomes or genomes for which isoform information was available (*Biomphalaria glabrata* and *Octopus bimaculoides*), only the longest isoform for each locus was retained. Proteins were clustered with OrthoFinder [52] at an inflation value of 3.0 using BLAST similarity information.

A total of 5 one-to-one single-copy orthologue clusters and 2182 ‘fuzzy’ single-copy orthologue clusters (clusters with maximum 5 proteins per taxon and with members in at least 75% of the taxa) were identified via KinFin [53]. Sequences in these 2187 clusters were aligned with MAFFT [48]. Alignments were trimmed with trimAl [49] and single-gene trees generated via RAxML [54]. Strict orthologues were inferred via PhyloTreePruner [55], yielding 1238 orthologous loci. Alignments of these were concatenated into a supermatrix of 262 970 distinct alignment positions (with 20.31% missing data) with FASconCAT [56] and a phylogenetic tree was inferred using RAxML [54].

An extended KinFin analysis was performed on the orthogroups in order to identify synapomorphic clusters and explore the expansions and contractions of protein families. KinFin defines synapomorphic clusters for each node of the phylogenetic tree using Dollo parsimony, which requires that only proteins of taxa under a given node be members of the cluster, and that proteins of at least one taxon from each child node be present. The topology of the phylogenetic tree of the taxa and the functional annotation of the proteins were supplied as input. For the KinFin analyses, taxa were grouped into the following taxonomic sets: Polyplacophora (*Acanthochitona crinita*), Gastropoda (*Biomphalaria glabrata*, *Lottia gigantea*, *Lymnaea stagnalis*), Bivalvia (*Bathymodiolus platifrons*, *Crassostrea gigas*, *Cristaria plicata*, *Laternula elliptica*, *Mya arenaria*, *Mytilus edulis*, *Mytilus galloprovincialis*, *Modiolus philippinarum*, *Mya truncata*, *Pecten maximus*), Solenogastres (*Gymnomenia pellucida*, *Wirenia argentea*), Scaphopoda (*Gadila tolmiei*), Monoplacophora (*Laevipilina hyalina*), Cephalopoda (*Octopus bimaculoides*, *Octopoteuthis deletron*, *Vampyroteuthis infernalis*), Caudofoveata (*Scutopus ventrolineatus*) and the outgroup (the annelid *Capitella teleta*).

Shell matrix proteins (SMPs) were identified by sequence similarity to a list of experimentally validated SMPs [57]. Proteins shorter than 50 residues were filtered out. Reciprocal best BLAST hits between SMPs and proteins in the clustering were evaluated via rbbh.py (https://github.com/DRL/rbbh).

## 3. Results and discussion

### (a) MolluscDB resources

MolluscDB is available openly at https://molluscdb.org. The database collates the genomic data for 22 species of mollusc including 7 species represented by genome sequences and 15 by transcriptome assemblies. Data are stored in the Ensembl schema and thus could be queried with tools developed by the Ensembl project [5], or custom tools using the Ensembl application programming interface (API). The 22 species in MolluscDB represent eight major classes of molluscs (table 1). For each assembly, a landing page includes a brief description of the species, information on the assembly and its annotation, and interactive plots displaying assembly metrics [58] and codon usage [59].

The GenomeHubs [12] implementation of the Ensembl infrastructure permits the hosting of multiple assemblies from the same species as well as multiple annotations of one assembly. Thus, for *L. stagnalis*, two annotations of the same assembly can be accessed. A text search function allows the discovery of specific sequences, regions or annotation terms. For each species genome, a gbrowse genome browser representation of the data is available. For the transcriptomes, the data are represented as one contig per assembled transcript, with the open reading frame annotated. Importantly, the annotation of genome-derived and transcriptome-derived proteins is consistent across species, as all the genomes and transcriptomes were decorated with functional inferences derived from searches and comparisons to the same libraries of reference information. We note that the assembled transcriptomes contain many more contigs (assembled transcripts) and have much longer spans than would be expected of a mollusc genome, and many more than the well-annotated complete genome sequences. This feature of transcriptome assemblies is well known, and results from a preponderance of short contigs supported by very few sequences. We have included the complete assemblies rather than filter by coverage as we would rather not exclude possibly biologically meaningful information.

MolluscDB includes an instance of Sequenceserver [13] so that a user can perform BLAST searches against any sequence hosted in the database at https://blast.molluscdb.org/. Two types of BLAST databases are available: nucleotide databases, which include scaffolds, transcripts and coding sequences (CDS), and protein databases. The BLAST search parameters can be modified from default to facilitate advanced search. A link in the header of every result connects each sequence with its Ensembl entry. The MolluscDB download server at https://download.molluscdb.org/ allows users to download any sequence or analysis hosted in the database. Files are consistently named and formatted.

### (b) Using MolluscDB to explore protein family evolution in Mollusca

**Figure 1.**
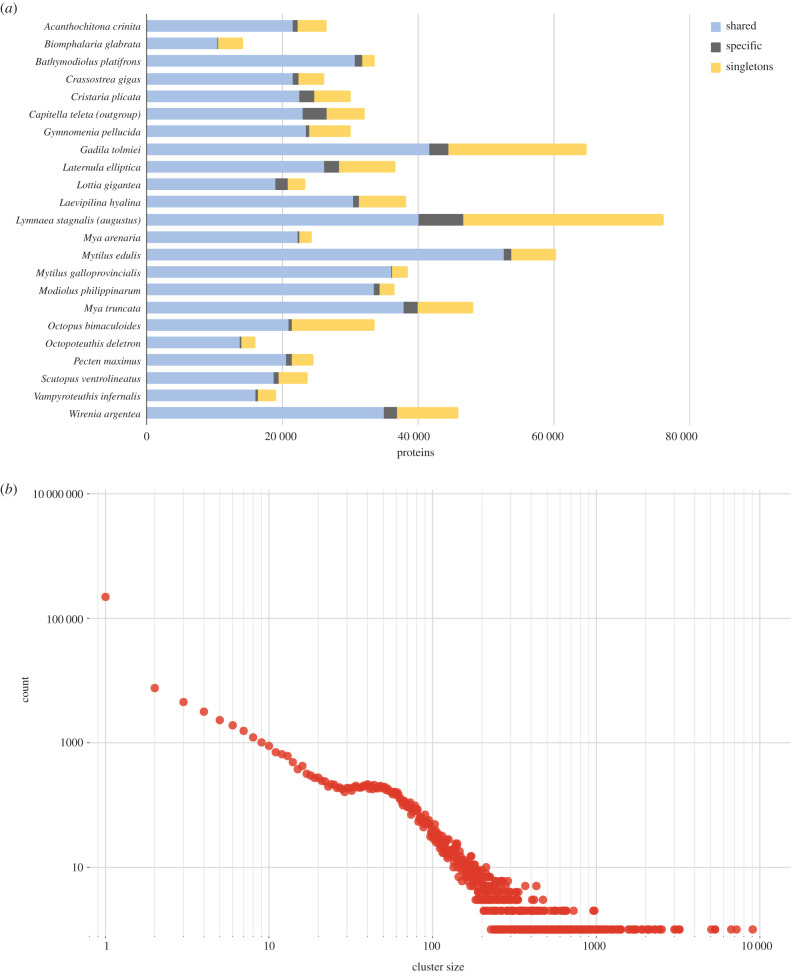
Protein families in species represented in MolluscDB. (*a*) Stacked histogram of proteins in each taxon analysed assigned to: ‘shared’: proteins in clusters containing proteins from multiple taxa; ‘specific’: proteins in clusters containing two or more proteins from a single proteome; and ‘singleton’: proteins in single protein clusters. (*b*) Frequency plot of cluster size in the OrthoFinder clustering of 214 608 orthogroups. (Online version in colour.)

We used an orthology clustering of the protein-coding genes in MolluscDB to explore the protein family traits of these species. A total of 802 455 proteins were retrieved after the filtering of the 23 proteomes included in the study. OrthoFinder [52] grouped these proteins into 214 608 clusters, 153 141 (71.4%) of which were singletons. *L. stagnalis* and *G. tolmiei* contributed the largest number of proteins to the clustering, and together they accounted for 32.62% of singleton clusters (figure 1*a*). The clusters presented a power-law-like frequency distribution with a deviation at cluster size 23 (figure 1*b*). Similar patterns have been observed before in such analyses [53,60–62]. This peak is largely made up of single-copy orthologues.

**Figure 2.**
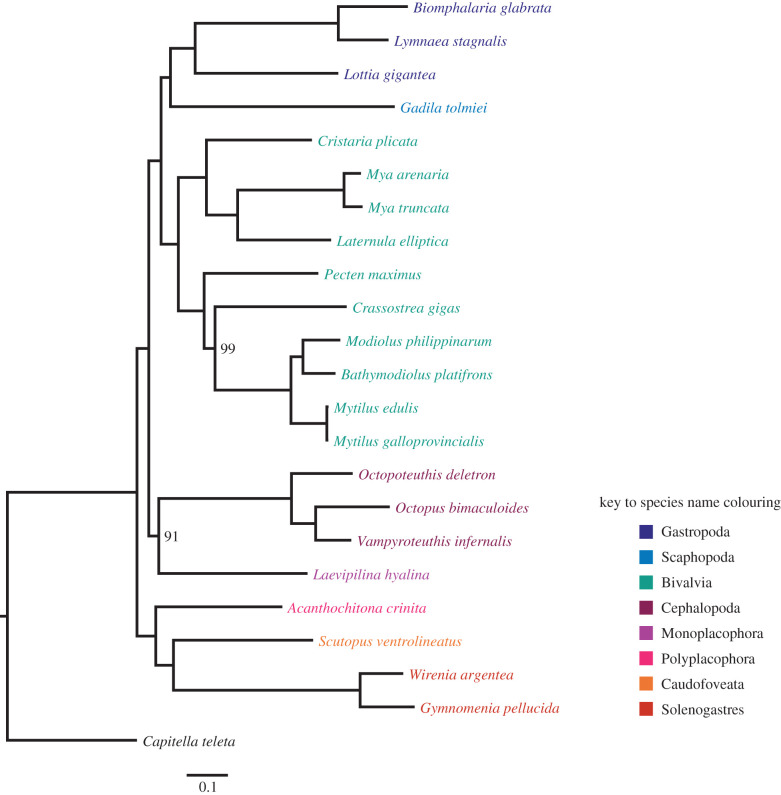
Phylogenetic tree of taxa in MolluscDB. Multilocus phylogeny of the species analysed. Support is 100 at all nodes except indicated.

A well-supported phylogeny was inferred from the set of 1238 orthologous loci (figure 2). All branches display bootstrap support values of 100, with the exception of two: the branch leading to Cephalopoda and Monoplacophora, and a branch leading to a subclade of Bivalves. Our results support a division in two major clades: Aculifera (Caudofoveata, Polyplacophora and Solenogastres) and Conchifera (Bivalvia, Cephalopoda, Gastropoda, Monoplacophora and Scaphopoda). Within Aculifera, Polyplacophora was placed sister to aplacophorans (Caudofoveata and Solenogastres). Within Conchifera, we recovered Bivalvia sister to a clade including Gastropoda and Scaphopoda. These results coincide with previous phylogenetic analyses [41,63]. Monoplacophora was recovered as the sister taxon of Cephalopoda. While this is consistent with Smith *et al.* [41], a recent study including genomic data from a newly sequenced monoplacophoran suggests that Monoplacophora could be a sister taxon to the rest of Conchifera [63]. Clearly, a more exhaustive sampling of loci across and within taxa is needed to create a robust phylogenetic framework for molluscs.

An insight on the distinctive biology of a monophyletic group can be gained through the study of synapomorphies. Just like morphological traits, molecular features such as gene presence and absence or gene family expansion and contraction are informative in a phylogenetic context. Using KinFin [53], we identified protein families that were unique to particular clades. By decorating these with functional attributes based on domain and sequence similarity, we assigned likely biological meaning to these synapomorphies. Here, we present an analysis of species in Bivalvia compared with all other mollusc groups in the database (table 2 and electronic supplementary material, table S2). Analysis of the OrthoFinder [52] clustering identified 14 synapomorphic clusters with the presence of at least five of the ten bivalve species. Annotations associated with these 14 clusters could be grouped into two main classes: immunity and metabolism. The dominant annotations related to immunity to cellular and viral pathogens, including RNA-dependent RNA polymerase (involved in the RNAi response to invading viral nucleic acids), big defensin [64,65], serum amyloid A [66,67], thaumatin [68], macin [69,70] and an immunity-related GTPase. Four Bivalvia-restricted clusters were annotated only as containing matches to domains of unknown function (DUF). One of these domains, DUF3421, has been associated with stress-responsive, sugar-binding natterins in *C. gigas* [71], where they may play roles in immune defence [72,73]. Metabolic annotations included carbohydrate metabolism (glycoside hydrolase family 76, and, possibly, a STELLO-like domain-containing protein), protein metabolism (peptidase S51) and degradation of vitamin B1 (Thiaminase-2/PQQC).

**Table 2.** Protein families that constitute molecular synapomorphies for Bivalvia. An extended version of this table is available in electronic supplementary material, table S2.

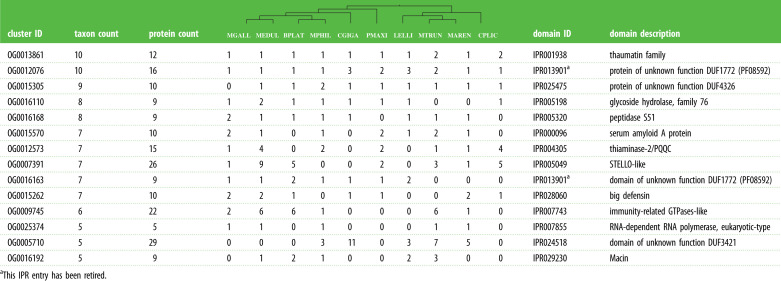

KinFin also facilitates analysis of expansion and contraction of protein families between clades by considering cluster membership count variation in a statistical framework akin to that deployed for gene expression analysis [53]. We identified protein clusters that had significantly different numbers of members in Bivalvia species than in the other taxa analysed (table 3 and electronic supplementary material, table S3). To be selected, the clusters had to have members in at least 7 Bivalvia and 7 other taxa, with Log2 mean ≥ 2 or ≤−2 and a *p*-value below 0.05 for the difference between Bivalvia and other taxa. There were 14 clusters with differential representation in Bivalvia. Twelve had higher family sizes in the bivalves. These included one annotated as tyrosinase, an enzyme implicated in shell formation [74–76], and several families annotated with domains associated with mollusc immunity, including C1q-like proteins [77], Toll/interleukin-1 receptor (TIR) [78] and fibronectin type III [79] domains. These findings mesh with previous descriptions of gene family expansions in bivalves of tyrosinase [80], C1q [77] and TIR [81]. Other enriched clusters have annotations including zinc-finger domains (C2CH-type and RING-type), carboxylesterase type B, neurotransmitter-gated ion-channel ligand-binding and transmembrane domains, TROVE domain, NIDO domain, BTB/POZ domain and a domain of unknown function (DUF229). Two clusters, annotated as containing peptidase M12A and LicD nucleotidyltransferase superfamily members, displayed a significantly lower number of proteins in Bivalvia compared with the other taxa.

**Table 3.** Protein families that have significantly different numbers of members in Bivalvia. An extended version of this table is available in electronic supplementary material, table S3.

cluster ID	species with members in cluster	total proteins in cluster	Bivalvia proteins in cluster	status	domain ID	domain description
OG0000078	22	241	189	enriched	IPR002227	tyrosinase copper-binding domain
OG0000280	22	123	97	enriched	IPR008858	TROVE domain
OG0000236	19	134	116	enriched	IPR001073	C1q domain
OG0000462	19	92	73	enriched	IPR006612	zinc finger, C2CH-type
OG0000931	19	63	51	enriched	IPR003886	NIDO domain
OG0000091	18	227	198	enriched	IPR027370	RING-type zinc-finger, LisH dimerization motif
OG0000121	18	198	186	enriched	IPR001611 IPR000157	leucine-rich repeat toll/interleukin-1 receptor homology (TIR) domain
OG0000216	18	142	119	enriched	IPR002018	carboxylesterase, type B
OG0000166	17	163	142	enriched	IPR006202 IPR006029	neurotransmitter-gated ion-channel ligand-binding domain neurotransmitter-gated ion-channel transmembrane domain
OG0000173	17	159	144	enriched	IPR000210 IPR011705	BTB/POZ domain BTB/Kelch-associated
OG0000607	17	79	68	enriched	IPR004245	protein of unknown function DUF229
OG0000275	15	123	107	enriched	IPR003961	fibronectin type III
OG0000451	18	94	8	depleted	IPR007074	LicD family
OG0000752	18	70	7	depleted	IPR001506	peptidase M12A

### (c) Shell matrix proteins

SMPs comprise a heterogeneous set of enzymes and structural proteins implicated in the biomineralization process, either by the demonstration of secretion by the mantle during shell synthesis or repair, or through isolation of peptides from isolated shell material [82]. We used a previously curated list of experimentally validated SMPs [57] to interrogate the gene sets in MolluscDB to identify presence/absence and gene family size change patterns (table 4 and electronic supplementary material, table S4).

**Table 4.** Functionally annotated shell matrix proteins. An extended version of this table is available in electronic supplementary material, table S4.

cluster ID	domain ID	domain description	number of proteins in cluster	number of species represented in cluster
OG0000137	IPR001223 IPR002557	glycoside hydrolase family 18, catalytic domain chitin-binding domain	182	23
OG0000159	IPR001466	beta-lactamase-related	167	23
OG0000231	IPR000668 IPR013201	peptidase C1A, papain C-terminal cathepsin propeptide inhibitor domain (I29)	136	23
OG0000631	IPR019479 IPR000866	peroxiredoxin, C-terminal alkyl hydroperoxide reductase subunit C/ thiolspecific antioxidant	77	23
OG0000962	IPR002130	cyclophilin-type peptidyl-prolyl *cis*-*trans* isomerase domain	62	23
OG0001572	IPR000741	fructose-bisphosphate aldolase, class-I	49	23
OG0001810	IPR017868 IPR001715	filamin/ABP280 repeat-like calponin homology domain	46	23
OG0000078	IPR002227	tyrosinase copper-binding domain	241	22
OG0000127	IPR019791	haem peroxidase, animal type	190	22
OG0000344	IPR015798 IPR015800	copper amine oxidase, C-terminal copper amine oxidase, N2-terminal	110	22
OG0002836	IPR001660	sterile alpha motif domain	38	22
OG0005847	IPR001715	calponin homology domain	28	22
OG0000686	IPR014044	CAP domain	73	21
OG0009156	IPR001152	beta-thymosin	23	21
OG0000036	IPR001304	C-type lectin-like	369	19
OG0000093	IPR002557 IPR002035	chitin-binding domain von Willebrand factor, type A	225	19
OG0000222	IPR031569	apextrin, C-terminal domain	138	19
OG0001850	IPR002937	amine oxidase	45	12
OG0011795	IPR000867	insulin-like growth factor-binding protein, IGFBP	17	10
OG0013954	IPR001223	glycoside hydrolase family 18, catalytic domain	12	8
OG0015275	IPR003961	fibronectin type III	10	7
OG0017162	IPR002035	von Willebrand factor, type A	8	7
OG0005249	IPR002035	von Willebrand factor, type A	30	6
OG0018497	IPR002223	pancreatic trypsin inhibitor Kunitz domain	7	6
OG0018680	IPR001148	alpha carbonic anhydrase	7	3
OG0020309	IPR015882 IPR015883 IPR004866	beta-hexosaminidase, bacteial type, N-terminal glycoside hydrolase family 20, catalytic domain chitobiase/beta-hexosaminidases, N-terminal domain	6	6
OG0020490	IPR001073	C1q domain	6	3

Seventy-four clusters presented proteins with significant sequence similarity to SMPs. Of the 48 clusters with three or more species, 27 had annotation matches to InterPro domains. These included highly conserved biomineralization domains such as tyrosinase, carbonic anhydrase, chitin-binding, von Willebrand factor, protease inhibitors and peroxidases [83,84]. We also retrieved proteins and domains associated with immune functions (C1q, fibronectin type III, C-type lectin and apextrin) [77,79,85–87]. Other matching sequences included proteins involved in metabolism (peptidase, fructose aldolase, lactamase, beta-hexosaminidase, oxidases and glycosyl hydrolases), cross-linking (filamin and calponin), protein folding (cyclophilin), actin filament organization (beta-thymosin) and regulation of insulin-like growth factors. These protein families are a strong substrate for future analysis of molecular correlates of mollusc responses to ocean acidification and warming, and for monitoring farmers' shellfish growth, health and disease.

### (d) Outlook

By collating genome and transcriptome data in a single database structure, we have been able to explore genomic data for diverse species of molluscs, and identify genes that may have evolved to deliver clade-specific processes. Using GenomeHubs [12] technology, we were able to incorporate genomes from both existing Ensembl instances and genomes that were too fragmented to incorporate in such pan-taxonomic databases. Transcriptome datasets are particularly attractive and economic to generate, as they sample only the expressed genome and allow immediate access to potential genes of interest. We have shown that these data can be rapidly incorporated and coordinated with full genome data in a consistent and accessible way. It is essential to recognize the key differences between transcriptome assembly-derived and genome-derived protein sets, such as the presence of multiple distinct isoforms and gene fragments in transcriptome assemblies. Despite this, the transcriptomes reliably report on the presence of a gene or gene family in a species, and facilitate filtering of lists of target genes to include (or exclude) those with broad phylogenetic representation.

MolluscDB currently presents 22 genomes and transcriptomes from the phylum Mollusca. To date, there are 49 genome assemblies in the International Nucleotide Sequence Database Consortium (INSDC; GenBank, European Nucleotide Archive, DNA Databank of Japan) databases (see https://www.ncbi.nlm.nih.gov/genome/?term=txid6447[Organism:exp], sourced 01 September 2020) with very different completeness and contiguity metrics. Incorporating these assemblies in MolluscDB is a near-future goal for the project. There are nearly 8000 mollusc transcriptome datasets in the short read archive (SRA) from 646 species (see https://www.ncbi.nlm.nih.gov/sra/?term=txid6447[Organism:exp]+and+transcriptomic, sourced 1 September 2020), 601 of which have no genome data. While some of these transcriptome datasets will not be suitable for assembly and presentation owing to low size or complexity of sample (including symbionts or other cobionts), they represent a large, currently untapped resource of information for comparative and functional genomics. Several global and regional projects, such as the Earth BioGenome Project [88] and Darwin Tree of Life project (https://darwintreeoflife.org), intend to sequence and assemble the genomes of large numbers of mollusc and other species, suggesting that the need for analysis hubs will only grow. Current database architectures may struggle to host and display such large amounts of data. For example, the 601 transcriptomes alone may generate 200 million assembled contigs and associated protein predictions and functional annotations. We are, therefore, also developing the GenomeHubs platform to scale to these new demands.

## Note added in proof

Since this article was accepted, Liu *et al.* have published their database of molluscan genome data, also called MolluscDB (at http://mgbase.qnlm.ac) (*Nucleic Acids Research* 2021, **49**: D1556. (Liu F, Li Y, Yu H, Zhang L, Hu J, Bao Z, Wang S. 2021 MolluscDB: an integrated functional and evolutionary genomics database for the hyper-diverse animal phylum Mollusca. *Nucleic Acids Research* **49**, D988—D997. (doi:10.1093/nar/gkaa918)). Their presentation includes similar functionality to MolluscDB presented here. We will liaise with the authors to ensure that the community is best served by our complementary efforts.

## Data accessibility

All data in MolluscDB are downloadable from the INSDC partner databases and/or from https://download.molluscdb.org/.
